# The Medical Officers of the State

**Published:** 1906-10

**Authors:** 


### EDITORIAL DEPARTMENT.

### THE MEDICAL OFFICERS OF THE STATE.

The policy of removing an experienced and efficient medical officer from any position without good and sufficient cause, is to be emphatically condemned. When it is done simply to make room for some personal or political favorite, or for some one in recognition of political services, it is simply outrageous. These positions should be filled by the ablest, best and most experienced men in the medical profession; and when the State has secured the right kind of men they should be retained by all means. Capable alienists are scarce. It requires long years of special study and experience to qualify a physician to deal with the insane; and such officer must combine also with his special knowledge an administrative and executive ability of no small degree. He must manage a big institution, very complicated in its machinery, with efficiency and economy. He must disburse large sums of mony appropriated by the State, and, in fact, do many things that require a special training and fitness. It is hard to find such men; and when they are found they should be retained indefinitely.

We have such men now, in charge of these great charities, and in the health department. It is manifestly to the best interest of the State, looked at from an economic standpoint, and beyond question, for the best interest of the insane population that few or no changes should be made in the management of these institutions. The insane are suspicious, and readily conceive a dislike to a person. They often have a suspicion that a certain person is an enemy who seeks their destruction; that is a characteristic delusion. They are easily excited, and the upsetting of an arrangement and a management to which they have become accustomed, the removal of familiar and friendly officers and employes has the same effect on them as stirring up a hornet's nest. It is not unattended with danger. The folly of such policy and the appointing to the superintendency of an insane asylum of a man inexperienced in such work and not acquainted with the characteristics and peculiarities of the insane, was horribly emphasized in the slaying of Superintendent Reeves some years ago by Purnell, an inmate of the asylum, who had been there many years, but was known to be dangerous, and was accordingly watched by former superintendents.

The experiment of putting in new men has been disastrous in other instances, and more than one resignation had to be asked for.

It stands to reason that no physician capable of filling such positions can afford to leave home and an established practice, break up and take such an office for two or four years. Hence we have had some ephemeral incompetents. In many States, most States, I believe, the superintendent and assistants have a life tenure of office.

Prior to the administration of Sayers, it was the policy to make a pretty clean sweep, by changing everything at the asylum, from superintendent to laundry women and servants. It was attended with discord and dissatisfaction. Sayers and Lanham had the good sense and wisdom to retain all who had proved themselves competent, and I am sure that it would be the part of wisdom, and no doubt Mr. Campbell will do it, to profit by past experience. The superintendent of our four insane asylums and their assistants have demonstrated their fitness for the position and are all men of long experience; men who have had their schooling in our great psychopathic hospitals. They have brought the institutions to a high state of efficiency and economy and they are a credit to Texas. In my deliberate judgment it would be exceedingly unwise to disturb them.

In thus emphatically expressing myself, I believe I voice the unanimous sentiment of the medical profession of the State.

